# Neurocritical Complications of Basal Cell Carcinoma With Intracranial Invasion

**DOI:** 10.7759/cureus.71080

**Published:** 2024-10-08

**Authors:** Katherine Silis, Matthew Hart, Chitra Sivasankar, Busra Delikkaya, Muhammad K Athar

**Affiliations:** 1 Neurocritical Care, Sidney Kimmel Medical College, Thomas Jefferson University, Philadelphia, USA; 2 Neurocritical Care, Thomas Jefferson University Hospital, Philadelphia, USA; 3 Pathology, Thomas Jefferson University Hospital, Philadelphia, USA

**Keywords:** basal cell carcinoma, critical care, intracranial invasion, neurologic sequelae, systemic complications of metastasis

## Abstract

Basal cell carcinoma (BCC) is the most common type of skin cancer, accounting for the majority of non-melanoma-type skin cancers. BCC is slow-growing and locally aggressive but rarely metastasizes.Although scarce, important consequences of untreated or recurrent BCC of the scalp are direct invasion into the skull, meninges, and/or brain. We describe a case of an adult male with a past medical history of basal cell nevus syndrome presenting with a BCC of the scalp complicated by intracranial extension. His critical condition resulted in complications including meningitis, seizures, and hypercoagulability, which ultimately resulted in the patient's death. This case reinforces the importance of routine follow-up as well as timely and aggressive treatment of skin cancer to prevent rare but severe systemic complications and neurologic sequelae.

## Introduction

Basal cell carcinoma (BCC) is the most common cause of skin cancer associated with UV radiation, often affecting sun-exposed areas including the head. It additionally has genetic risk factors [1]. One such genetic syndrome increasing the risk for BCC is Gorlin, or basal cell nevus syndrome, an autosomal dominant disease resulting from mutations in the PTCH1 gene. This syndrome clinically causes an increased risk for BCC, jaw cysts, papules with telangiectasias, medulloblastoma, and skeletal deformities [2]. While a slow-growing course is typical, progression to metastatic BCC is extremely rare with an incidence of 0.0028-0.55% [3]. BCC with direct intracranial extension is another important complication of severe or untreated BCC involving the scalp. When this occurs, these cases tend to have high morbidity and mortality [4]. Rare complications including seizures, hypercoagulability, and infection may be prevented by aggressive preventative care.

## Case presentation

**Figure 1:**
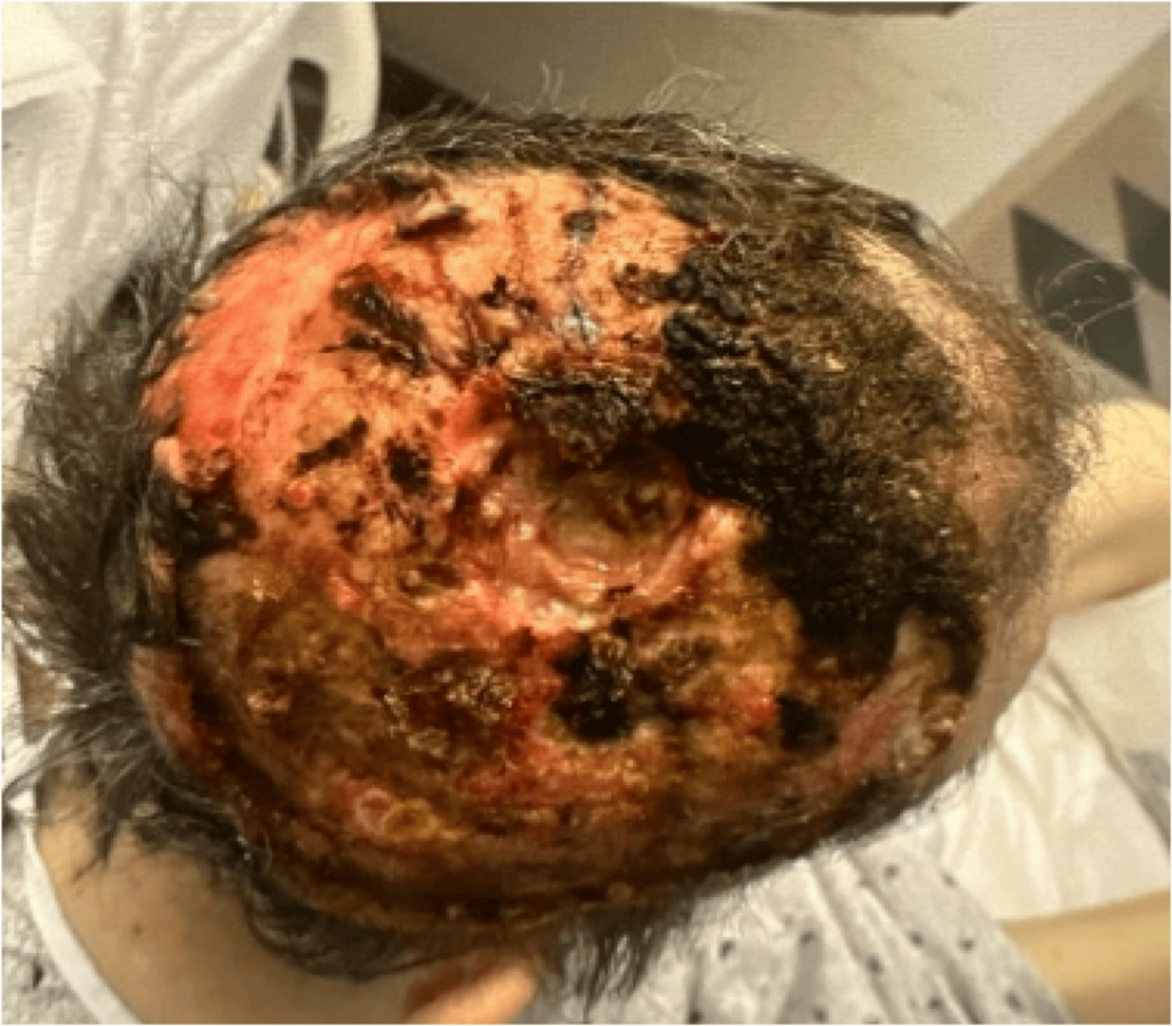
Skull defect on hospital admission

The patient was a 73-year-old male with a 35-year history of basal cell nevus syndrome presenting with an untreated ulcerated wound on his scalp secondary to BCC. He presented to the emergency department of a community hospital from a skilled nursing facility (SNF) for worsening scalp wounds (Figure 1), foul-smelling discharge, defect of the parietal bone, and forgetfulness. His only neurological symptom was encephalopathy, with no focal neurologic deficits on examination. Previously, this patient had received four Mohs surgeries over 16 years, the last occurring four years ago. Pathology at that time demonstrated BCC, nodular subtype. He presented at that time with a wound at the same site on the right scalp, which had enlarged since then. The wound was so extensive that the patient was instructed to follow up with plastic surgery and at that time moved to a SNF to assist with extensive wound care needs. This patient was following up with a surgical oncologist prior to hospitalization and recommended to start vismodegib, a Hedgehog inhibitor highly effective in basal cell nevus syndrome, but was lost to follow-up.

**Figure 2:**
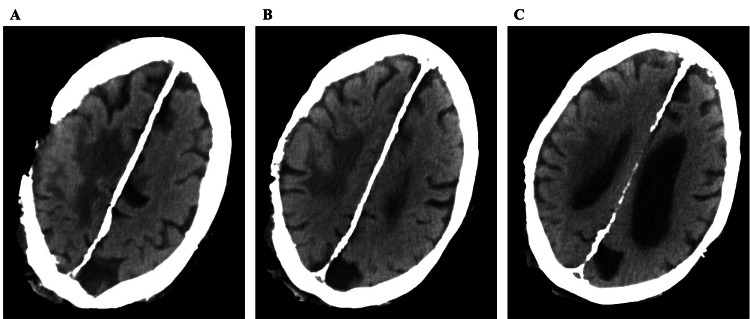
Non-contrast CT of the head demonstrates vasogenic edema secondary to invasive malignancy A through C demonstrate a right skull defect and a right frontoparietal hypodensity consistent with vasogenic edema at multiple descending levels of the CT scan; this was concerning for an erosive cancerous process. CT: computed tomography

**Figure 3:**
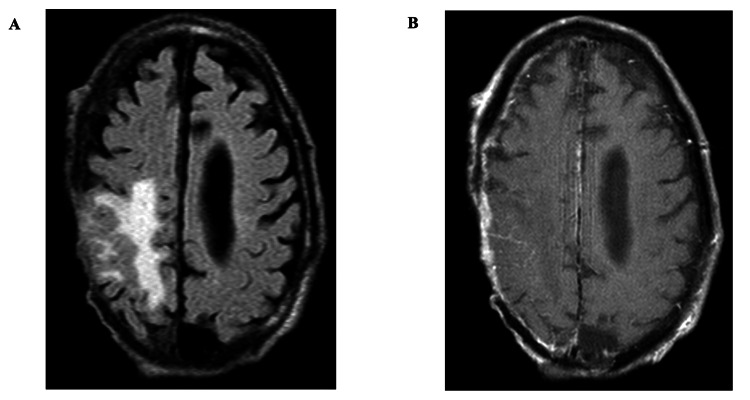
T2 FLAIR MRI shows pachymeningeal enhancement consistent with intracranial spread of basal cell carcinoma MRI with and without contrast demonstrates a T2 FLAIR hyperintensity in the right frontoparietal lobe and a right frontoparietal skull defect (A). This is consistent with vasogenic edema, with correlating right-sided pachymeningeal enhancement on T1 post-contrast sequence (B). T2 FLAIR: T2-weighted fluid-attenuated inversion recovery; MRI: magnetic resonance imaging; T1: T1-weighted MRI

The initial concern on hospitalization was for cellulitis, osteomyelitis, and sepsis. This resulted in patient admission for treatment with intravenous antibiotics including cefazolin and vancomycin. Computed tomography of the head (CTH) imaging revealed a large area of hypodensity within the right frontal and parietal lobes near the vertex and erosion of the skull (Figure 2A-2C). A subsequent magnetic resonance imaging of the brain (MRI-B) with and without contrast confirmed a right frontoparietal skull defect. This revealed a T2-weighted fluid-attenuated inversion recovery (T2 FLAIR) hyperintensity extending into the right frontal and parietal lobes with correlating pachymeningeal enhancement (Figure 3A, 3B). These neuroradiographic findings were consistent with cerebral edema, from a parenchymal lesion, skull erosion from his neoplastic process, and meningitis. The scalp defect was found to be bigger than the intracranial defect, crossing midline over the right frontal, parietal, and temporal regions into the left parietal scalp.

**Figure 4:**
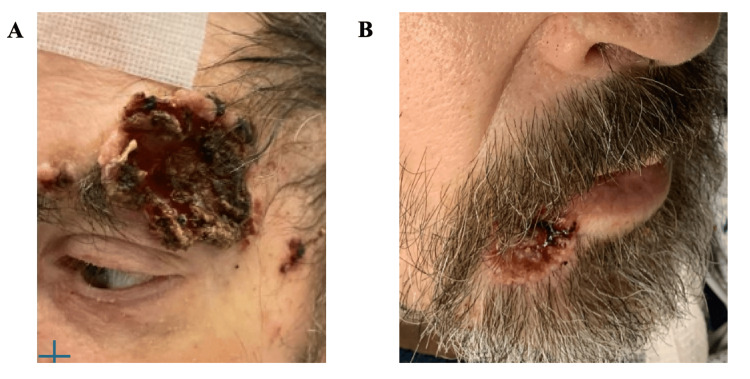
Skin lesions including the left forehead (A) and the right lower lip (B) documented at a quaternary care center

Due to the need for an interdisciplinary team including neurosurgery, oncology, and infectious disease, this patient was transferred from a community hospital to a quaternary care hospital where his scalp lesions were re-evaluated. On exam, the patient was found to have other skin lesions including above the left eye and below the right lower lip (Figure 4A, 4B).

**Figure 5:**
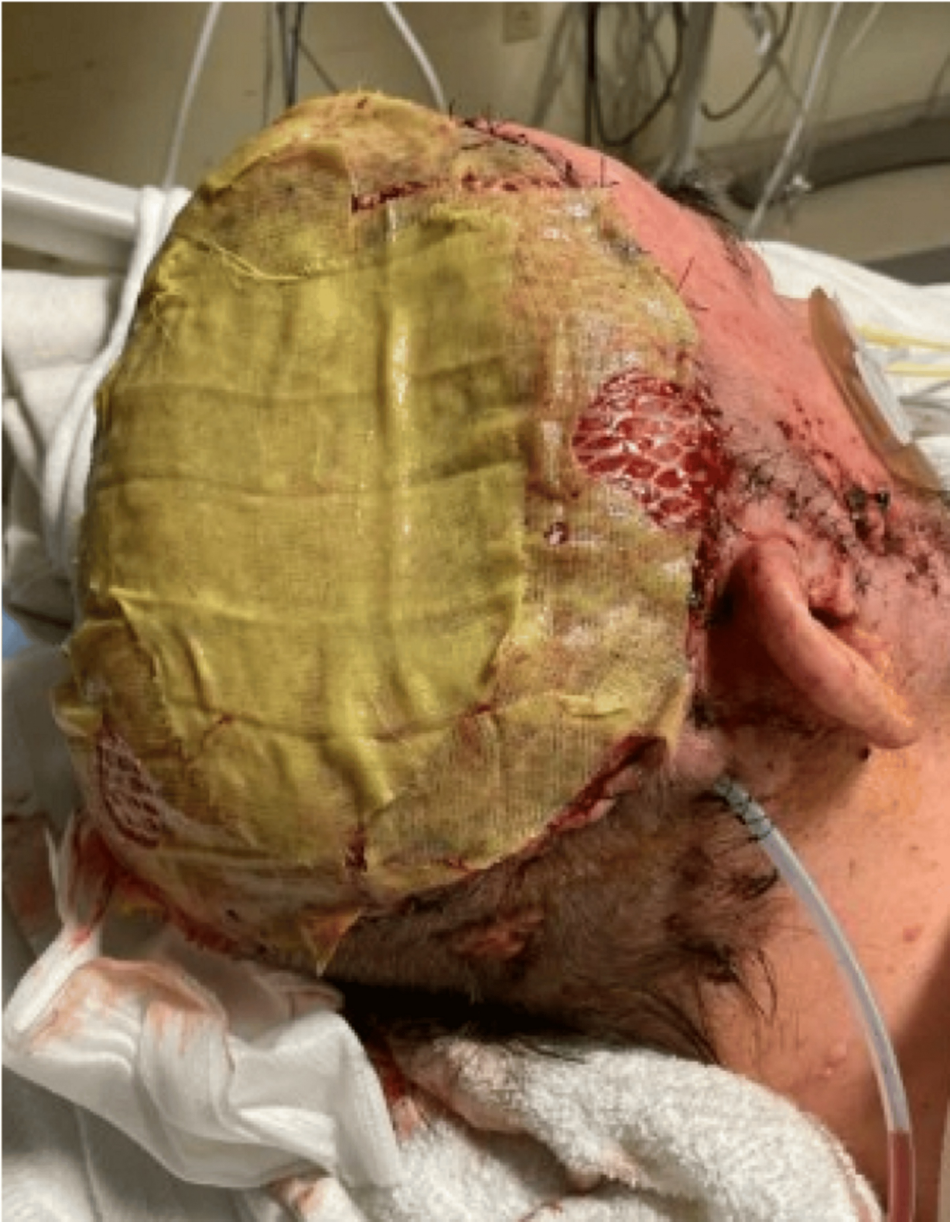
Postsurgical debridement with Integra bilayer matrix temporary graft and wound vacuum placement

**Figure 6:**
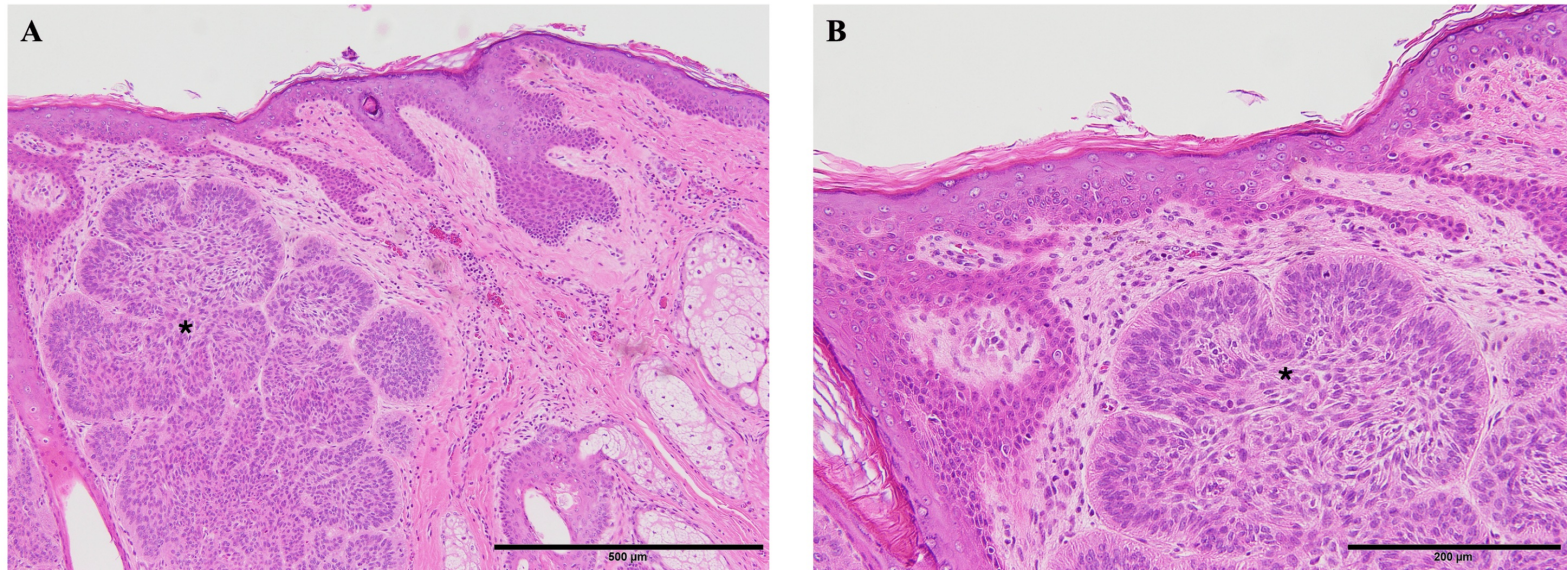
Pathology specimen of basal cell carcinoma (*) infiltrating the dermis (A) H&E staining; 10× magnification. (B) H&E staining; 20× magnification. H&E: hematoxylin and eosin stain

**Figure 7:**
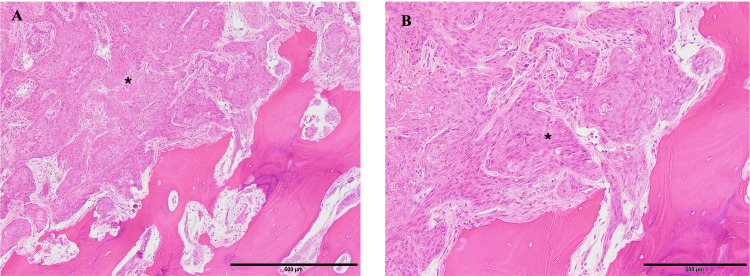
Pathology specimen of basal cell carcinoma (*) infiltrating the skull (A) H&E staining; 10× magnification. (B) H&E staining; 20× magnification. H&E: hematoxylin and eosin stain

**Figure 8:**
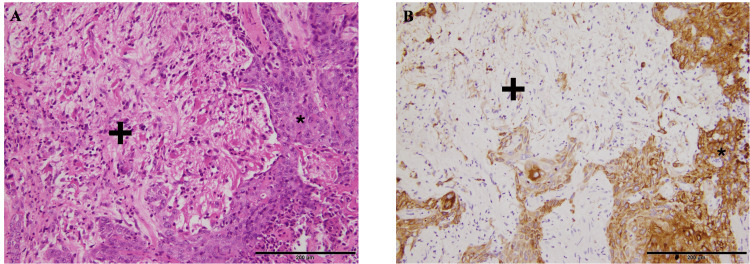
Pathology specimen of basal cell carcinoma (*) infiltrating the brain tissue (+) (A) H&E staining; 20× magnification. The tumor epithelial cells (*) show positive immunohistochemical staining for cytokeratin. (B) AE1/AE3 staining; 20× magnification. H&E: hematoxylin and eosin stain; AE1/AE3: antibody stain for epithelial cells

The patient underwent skull and scalp debridement, followed by craniectomy and neurosurgical intervention for mass resection with Integra bilayer matrix temporary graft and wound vacuum placement (Figure 5). Surgical pathology showed that cutaneous BCC had extended into the dermis (Figure 6A, 6B), skull (Figure 7A, 7B), and brain tissue (Figure 8A, 8B). This patient subsequently received plastic surgery for a latissimus dorsi flap with a thigh donor skin site and was extubated. During his hospitalization, the patient was treated with vancomycin, cefepime, metronidazole, and fluconazole. While he had negative blood cultures and *Clostridium difficile* polymerase chain reaction (PCR), his operating room (OR) cultures from brain specimens had polymicrobial results positive for *Proteus*, *Pseudomonas*, *Enterococcus*, and *Corynebacterium*. Post-operatively, the patient had persistent encephalopathy and new left-sided hemiparesis. Repeat CTH imaging was negative. He was empirically treated for seizure, which could not be confirmed by electroencephalogram (EEG) due to his cranial flap, with levetiracetam, lacosamide, and valproic acid. There was moderate improvement in the exam with anti-epileptic drugs. In an attempt to wean valproic acid to limit polypharmacy, the patient's mental status worsened, prompting its continuation. 

**Figure 9:**
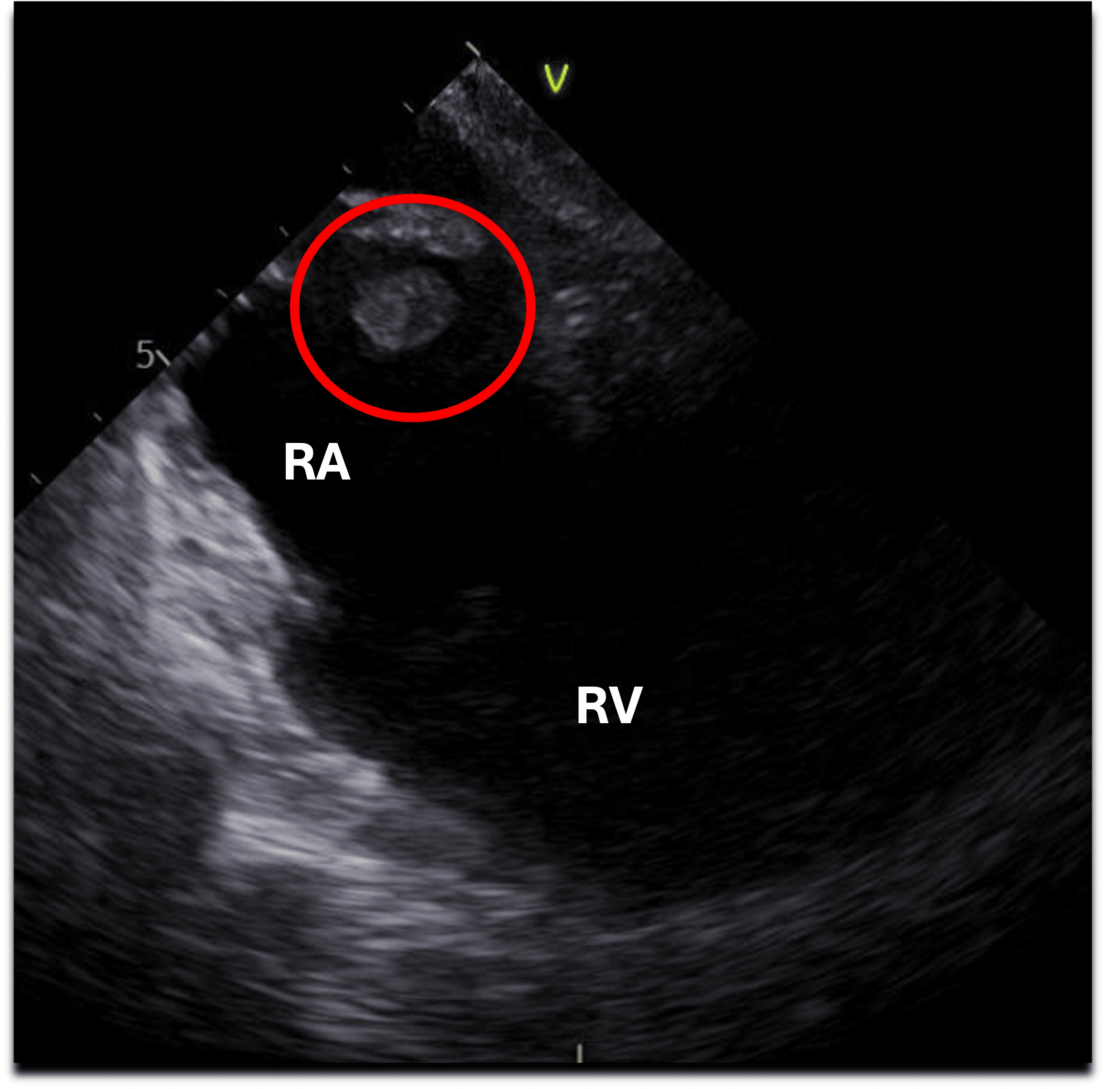
TEE consistent with right atrial thrombus (red circle) TEE: transesophageal echocardiogram; RA: right atrium; RV: right ventricle

**Figure 10:**
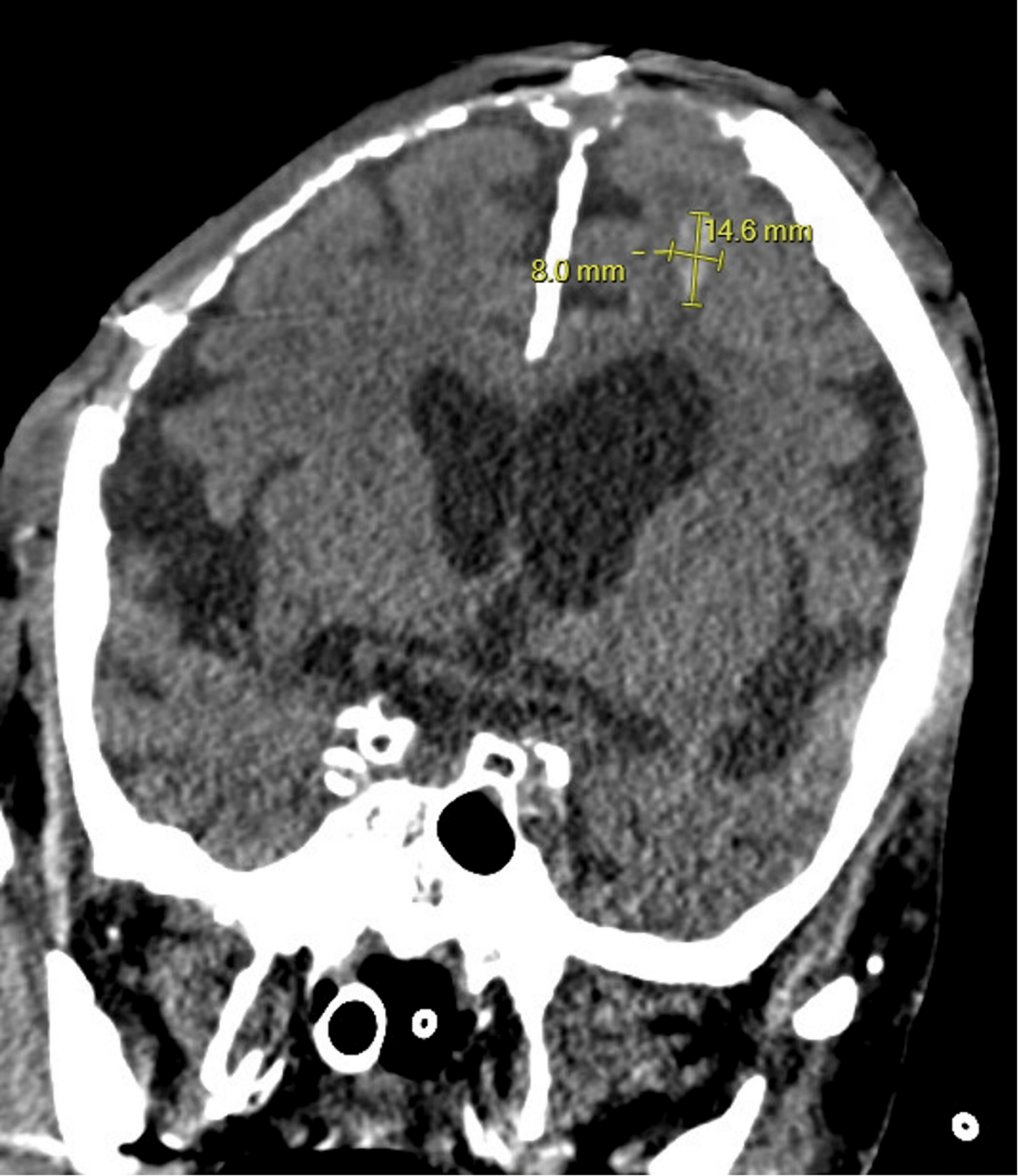
Coronal view of non-contrast CT of the head reveals intraparenchymal hemorrhage Repeat CT of the head demonstrating an 8×14.6 mm left frontal hyperdensity consistent with an intraparenchymal hemorrhage in the setting of heparin use for the treatment of the patient's right atrial thrombus and pulmonary emboli. This was stable on repeat imaging. CT: computed tomography

On post-operative day 5, the patient developed new bradycardia and hypotension. Lacosamide was transitioned to levetiracetam to limit the potential contribution to an arrhythmia. A transesophageal echocardiogram obtained demonstrated a right atrial thrombus (Figure 9), and subsequent CT angiogram of the chest revealed bilateral segmental and subsegmental pulmonary embolism and right atrial thrombus. He was managed with heparin. Out of concern for potential hemorrhagic transformation, a surveillance CTH was obtained when the patient had a therapeutic partial thromboplastin time. It exhibited a new small intracranial hemorrhage which was stable on repeat scan (Figure 10); thus, the heparin was continued.

**Figure 11:**
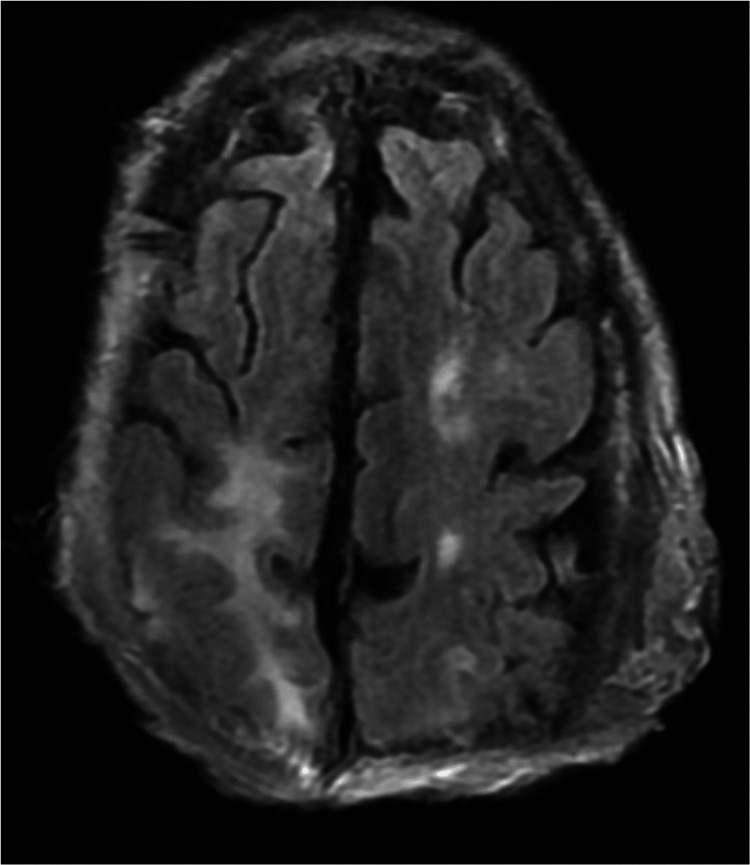
Postsurgical T2 FLAIR MRI reveals signs of embolic stroke and edema This image shows a right parietal T2 FLAIR hyperintensity consistent with postsurgical edema and left-sided hyperintensities consistent with embolic strokes seen on diffusion-weighted imaging. T2 FLAIR: T2-weighted fluid-attenuated inversion recovery; MRI magnetic resonance imaging

Unfortunately, the patient decompensated into worsening encephalopathy and hypoxic respiratory failure requiring intubation. Repeat MRI showed embolic strokes on diffusion-weighted imaging and persistent right parietal T2 FLAIR hyperintensity consistent with postsurgical edema (Figure 11), but no other significant findings to explain his degree of encephalopathy. He was later transitioned to comfort care. The patient passed away during hospitalization due to systemic neurocritical complications of BCC.

## Discussion

Cases of direct intracranial spread of BCC have been reported in literature including spread from the nose causing blindness and paralysis [5], invasion from the occipital bone to the cerebellum [6], skull base invasion with cranial nerve involvement [7], and frontal lobe invasion causing personality changes [8]. Most cases occurred in the setting of poor disease control in Caucasian patients and resulted in neurological deficits as precursors to workup including cranial nerve palsies and vision loss.

Our case uniquely had multiple systemic complications of metastatic disease including polymicrobial scalp wounds positive for gram-positive and gram-negative organisms, which is consistent with prior studies. In one study of chronic scalp wounds with delayed debridement from the Wenchuan earthquake in China, 64% of organisms were gram-positive (*Staphylococcus aureus* and *Staphylococcus epidermidis*) with 36% represented by gram-negatives such as *Enterobacter*, *Klebsiella*, and *Serratia* [9]. Prior studies have shown that biofilms in the setting of chronic wounds are the culprit of fulminant infection. Keys to diagnosis include procalcitonin and leukocyte count; management requires prompt debridement and antimicrobial therapy [10]. In this case, the patient had chronic scalp wounds in the setting of patient noncompliance with treatment and prior site of BCC surgery requiring repeat surgical intervention. 

This patient additionally had evidence of hypercoagulability in the setting of metastatic skin cancer and systemic involvement, which is the second leading cause of death in cancer patients and is most common in lung, stomach, colon, and breast cancer. The cause is multifactorial including prothrombotic factors released in the setting of cancer, stasis, and treatments including chemotherapy [11]. Studies investigating hypercoagulability in the setting of skin cancer are limited. One study of melanoma patients based on a national registry revealed that 5% had venous thromboembolism (VTE) while hospitalized, which was associated with morbidity and death [12]. Another study found keratinocyte carcinoma did not cause a significant increase in VTE [13].

While the gold standard of seizure diagnosis is video EEG, in this case, the patient could not have electrodes placed due to a lack of viable scalp in the setting of surgery. Seizure prophylaxis was initiated based on clinical suspicion with new, intermittent unilateral weakness and altered mental status concerning for seizures. While new devices including in-ear EEG are currently being researched [14], avenues of diagnosing seizures without scalp EEG other than clinical signs will be an important subject of future studies. 

One of this patient's risk factors for Gorlin syndrome is basal cell nevus syndrome, a form of genetic BCC with increased risk for multiple lesions responsive to medications which target the Sonic Hedgehog pathway. There are three documented cases of distally metastatic basal cell nevus syndrome; however, it is still poorly understood if Gorlin syndrome confers an increased risk of metastasis when compared to other forms of skin cancer [15].

## Conclusions

An adult male presented with a BCC of the scalp complicated by intracranial extension requiring neurosurgical intervention, skin grafting, and a lengthy hospital course ultimately resulting in demise. Notably, the patient did not present with primary neurologic deficits as an initial chief complaint, reinforcing that although BCC normally has an indolent and slow course, it is crucial to intervene to prevent rare forms of metastasis including direct intracranial spread. Complex neurocritical complications can be deadly in these cases. Patient education is critical to help reduce rates of treatment noncompliance like this and loss to follow-up.
